# The role of microRNAs in learning and long-term memory

**DOI:** 10.18699/VJ20.687

**Published:** 2020-12

**Authors:** L.N. Grinkevich

**Affiliations:** Pavlov Institute of Physiology of the Russian Academy of Sciences, St. Petersburg, Russia

**Keywords:** epigenetics, miRNA, learning, long-term memory, cognitive impairment, sleep deprivation, environmental enrichment, эпигенетика, микроРНК, долговременная память, когнитивные нарушения, депривация сна, обогащенная среда

## Abstract

The mechanisms of long-term memory formation and ways to improve it (in the case of its impairment) remain an extremely difficult problem yet to be solved. Over the recent years, much attention has been paid to microRNAs in this regard. MicroRNAs are unique endogenous non-coding RNAs about 22 nucleotides in length; each can regulate translation of hundreds of messenger RNA targets, thereby controlling entire gene networks. MicroRNAs are widely represented in the central nervous system. A large number of studies are currently being conducted to investigate the role of microRNAs in the brain functioning. A number of microRNAs have been shown to be involved in the process of synaptic plasticity, as well as in the long-term memory formation. Disruption of microRNA biogenesis leads to significant cognitive dysfunctions. Moreover, impaired microRNA biogenesis is one of the causes of the pathogenesis of mental disorders, neurodegenerative illnesses and senile dementia, which are often accompanied by deterioration in the learning ability and by memory impairment. Optimistic predictions are made that microRNAs can be used as targets for therapeutic treatment and for diagnosing the above pathologies. The importance of applications related to microRNAs significantly raises interest in studying their functions in the brain. Thus, this review is focused on the role of microRNAs in cognitive processes. It describes microRNA biogenesis and the role of miRNAs in the regulation of gene expression, as well as the latest achievements in studying the functional role of microRNAs in learning and in long-term memory formation, depending on the activation or inhibition of their expression. The review presents summarized data on the effect of impaired microRNA biogenesis on long-term memory formation, including those associated with sleep deprivation. In addition, analysis is provided of the current literature related to the prospects of improving cognitive processes by influencing microRNA biogenesis via the use of CRISPR/Cas9 technologies and active mental and physical exercises.

## Abstract (ru)

Механизмы формирования долговременной памяти и способы ее улучшения (в случае нарушения) остаются сложнейшей нерешенной проблемой. В последние годы большое внимание в этой связи уделяется микроРНК. МикроРНК являются уникальными эндогенными некодирующими РНК длиной около 22 нуклеотидов, каждая из которых может регулировать трансляцию сотен матричных РНК, тем самым управляя целыми сетями генов. МикроРНК широко представлены в центральной нервной системе. В настоящее время значительное количество исследований посвящено изучению роли микроРНК в функционировании мозга. Показано, что целый ряд микроРНК вовлечен в процесс синаптической пластичности, а также в формирование долговременной памяти. При этом нарушение биогенеза микроРНК приводит к значительным когнитивным дисфункциям. Более того, нарушение биогенеза микроРНК является одной из причин патогенеза заболеваний, связанных с психическими расстройствами, нейродегенеративными патологиями и старческой деменцией, которые часто сопровождаются ухудшением способности к обучению и нарушением памяти. Высказываются оптимистичные прогнозы, что микроРНК могут быть использованы в качестве мишеней для терапевтического лечения и диагностики данных патологий. Важное прикладное значение микроРНК увеличивает интерес к изучению их функций в работе мозга. Представленный обзор посвящен роли микроРНК в когнитивных процессах. Описаны биогенез микроРНК и роль микроРНК в регуляции экспрессии генов. Рассмотрены последние достижения в изучении функциональной роли микроРНК в обучении и формировании долговременной памяти, в зависимости от активации или ингибирования их экспрессии, и о влиянии нарушения биогенеза микроРНК на формирование долговременной памяти. Небольшой раздел посвящен влиянию депривации сна на когнитивные процессы, зависимые от микроРНК. Кроме того, приведен анализ текущей литературы, связанной с перспективами улучшения когнитивных функций посредством влияния на биогенез микроРНК путем применения CRISPR/Cas9 технологий и активных умственных и физических нагрузок

## Introduction

The mechanisms of forming long-term memory (LTM) and of its improvement in case of impairment resulting from trauma, neurological and neurodegenerative diseases, and age-related dysfunctions are among the most challenging issues to be solved by the science. Back in the middle of the last century, researchers realized that LTM formation requires active involvement of the genome. Later, it was shown that newly synthesized proteins are necessary for modification of synaptic contacts and rearrangements of the neural networks involved in consolidating new experiences (Sweatt, 2016). The main difficulties in studying the molecular basis of LTM are associated with both complexity of the structure of the central nervous system and with variety of the regulatory processes acting at the genome level. The latter include regulation of gene expression by DNAbinding transcription factors, as well as epigenetic modifications that regulate the structure of chromatin (Berger, 2007). These epigenetic processes are widely involved in brain functioning, including neuronal differentiation and adaptive behavior, inter alia LTM formation (Fischer, 2014; Kim, Kaang, 2017).

Somewhat later, studies were started on involvement of microRNA in the epigenetic regulation of gene expression. MicroRNAs are unique non-coding molecules, each of which able to regulate translation of hundreds to thousands of messenger RNAs (mRNAs) targets. MicroRNAs are most widely represented in the central nervous system, and many of them are expressed at a high level (Chen, Qin, 2015). A large number of studies have been published on microRNAs participation in neuronal differentiation (Baek et al., 2014; Chen, Qin, 2015), in the LTM formation (Rajasethupathy et al., 2009; Gao et al., 2010; Hu Z., Li, 2017), as well as in the pathogenesis of diseases associated with mental disorders, neurodegenerative pathologies and senile dementia (Beveridge et al., 2010; Danka Mohammed et al., 2017; Wingo et al., 2020). Optimistic predictions are expressed that a number of microRNAs can be used as targets for therapeutic treatment and diagnosis of diseases accompanied by cognitive impairment (Liu et al., 2017). This important applied value of microRNAs raises interest in the study of their functions. This review will examine in detail the role of microRNAs in cognitive processes.

## MicroRNAs – biogenesis and mechanism of regulating gene expression

For the first time, microRNAs, as functionally significant molecules capable of regulating gene expression, were described in the nematode C. elegans in 1993 (Lee R. et al., 1993). In 2000, highly conserved microRNA let-7, which is necessary for organism development, was discovered in the same animal (Reinhart et al., 2000). In parallel, in 1998, A. Fire and C. Mello published an article in which it was shown that by means of small double-stranded RNAs (siRNA) it is possible to silence genes – this mechanism was called RNA interference. Moreover, already in 2006, in view of the importance of this discovery, Andrew Fire and Craig Mello were awarded the Nobel Prize in Physiology or Medicine. It has been shown that gene silencing by means of RNA interference is also carried out by microRNA (He, Hannon, 2004). Since the 2000s, an avalanche of studies on the functional role of microRNA and its biogenesis in many animal species started

MicroRNAs are a family of small, highly conserved endogenous non-coding RNAs about 22 nucleotides long (He, Hannon, 2004; Bitetti et al., 2018). MicroRNA biogenesis is a complex and multistep process, including transcription from DNA of a rather long primary transcript (pri-miRNA) with characteristic stem-loop structures, its processing to form pre-miRNA, translocation of pre-miRNA into the cytoplasm and its further processing to form microRNA (miRNA). Next, the microRNA interacts with the RISC complex (RNA-induced silencing complex) in which the microRNA binds to the target mRNA and induces degradation and/or mRNA translational repression (Bartel, 2009; Aksoy-Aksel et al., 2014). Evolutionarily conserved proteins control all of the above stages: DROSHA, DGCR8, EXP5, RAN, DICER, TARBP2, AGO, and PIWI. Proteins DROSHA and DGCR8 are endonucleases and control the processing of the primary microRNA transcript. EXP5 and RAN are involved in translocation of pre-miRNAs from the nucleus to the cytoplasm. DICER endonuclease regulates cleavage of pre-miRNAs to form mature miRNAs and association of mature miRNAs with the RISC. The current data on microRNA biogenesis are described in detail in the review by Smith and Kenny (Smith, Kenny, 2018).

In the cytoplasm of neuronal cell bodies, miRNAs are often associated with processing structures that are responsible for storage and degradation of mRNA, and can also be found in stress granules that are formed in response to stress (Leung, 2015; Smith, Kenny, 2018). Expression of miRNAs in neurons is induced by electrical activity and downstream regulatory cascades at several levels, including the synthesis and processing of the primary transcript, processing of premiRNAs, and assembly of the RISC (Aksoy-Aksel et al., 2014). However, these processes are still poorly understood. MicroRNAs are highly stable molecules (up to 10 times more stable than mRNAs) (Gantier et al., 2011).

The complexity of studying the role of miRNAs in the brain is determined by the variety of neuronal and glial cells that perform different functions and express different patterns of miRNAs (McNeill, Van Vactor, 2012; Malmevik et al., 2016). Moreover, each miRNA can have hundreds of different mRNAs as targets, and expression of a particular mRNA can be regulated by several miRNAs (Lewis et al., 2003; John et al., 2004). Therefore, dysregulation of a single miRNA can have a large polygenic effect. The number of identified miRNAs already amounts to several thousands, and according to various estimates, miRNAs are capable of regulating the expression of 30 to 70 % of all genes encoding proteins at the posttranscriptional level (Selbach et al., 2008). It is important that about 70 % of miRNAs are expressed in the brain, and quite differentially in different regions (cited after Chen, Qin, 2015). In addition, microRNAs can be secreted into the extracellular space, including the circulatory system, to ensure intercellular and interorgan communication (Lesseur et al., 2014; Smith, Kenny, 2018). The pattern of extracellular miRNAs changes in a number of pathologies, and these data are beginning to be used for diagnostic purposes (Lesseur et al., 2014; Smith, Kenny, 2018). Thus, miRNAs are the key regulators of many gene networks and, accordingly, can coordinate the most important processes in the organism. 

MicroRNAs in learning and long-term memory

Currently, several learning models are used to study the molecular mechanisms of LTM, which can be divided into associative and non-associative ones. The former includes development of various conditioned reflexes, and the latter includes non-associative analogs of learning, such as sensitization (facilitation), depression (habituation), post-tetanic potentiation and post-tetanic depression.

Sensitization is “enhancement of a pre-existing response of an animal to a stimulus as a result of application of another, nociceptive (painful) stimulus”. Sensitization is necessary for an animal to respond to a stimulus that was previously insignificant for it (Kandel, 1982). Depression (habituation) is weakening of the response to a previously significant stimulus as a result of its periodic reiteration. Through habituation, an animal learns to ignore stimuli that have lost their novelty or meaning. At the cellular level, sensitization is associated with an increase in the efficiency of synaptic communication between neurons, and depression – with its weakening (Kandel, 1982).

Post-tetanic potentiation is improvement in synapse conduction after a series of frequent (tetanizing) stimulations of incoming fibers. Post-tetanic depression is deterioration in synaptic conduction after a series of weak rhythmic stimuli.

Long-term changes in the efficiency of synaptic transmission in these models require involvement of the genome and are caused by long-term plastic changes in the synapses through remodeling of presynaptic and/or postsynaptic structures, the spiny morphogenesis, and/or growth or elimination of synapses. Thus, synaptic plasticity is the mechanism by which the brain encodes and stores information. It is believed that these processes underlie LTM formation in both animals and humans. 

The role of microRNAs in synaptic plasticity and local biosynthesis in neurites

The role of microRNAs in synaptic plasticity has been most fully investigated using models of long-term post-tetanic depression (LTD) and post-tetanic potentiation (LTP). Local protein synthesis plays an important role in these processes. It has been shown that the synthesis of receptors, proteins involved in the transport of synaptic vesicles and proteins needed for modeling the growth of spines can occur in neurites, since neurites contain the necessary set for translation of mRNA, including ribosomes, and, moreover, pre-microRNA, microRNA and Dicer enzyme, which allows microRNA to regulate local biosynthesis (Lugli et al., 2005; Bicker et al., 2013; Smalheiser, 2014; Hu Z., Li, 2017). Thus, during LTD formation in the dendrites of hippocampal neurons, local synthesis of the glutamate receptor GluA1 occurs, its expression is regulated by miR-501-3p, and this process is required for remodeling of dendritic spines, the density of which determines the efficiency of the synapse (Hu Z. et al., 2015).

Long-term remodeling of the dendritic tree also involves miR-191, miR-135, and miR-137 (Hu Z. et al., 2014; Siegert et al., 2015). On the other hand, miR-26a and miR-384-5p participate in the formation of LTP, the expression of which decreases during tetanization in an RSK3-dependent manner. Structural and signaling proteins of synapses (Gu Q. et al., 2015) are the targets of these microRNAs. At the same time, LTP is accompanied by expansion of spines and formation of new ones, while the opposite picture is observed for LTD (Hu Z., Li, 2017). It has been suggested that modulation of protein synthesis in synapses may be based on local Ca2+-dependent activation of the Dicer enzyme (Lugli et al., 2005).

The effect of microRNA biogenesis disorders on LTM formation

In the initial studies of involvement of miRNAs in learning and memory, animals with genetic impairments of enzymes involved in miRNA biogenesis, in particular, with dysfunction of the Dicer, were used (Konopka et al., 2010; Fiorenza, Barco, 2016; Fiorenza et al., 2016). Induced by Tamoxifen injection, deletion of Dicer in the forebrain of mice (mutation Dicer1CaMKCreERT2) has been shown to cause the loss of a number of brain-enriched microRNAs, including miR-124, miR-132, miR-137, miR-138, miR-29a/c, and these mice show improved memory (Konopka et al., 2010). In animals with Dicer suppression, the excitability of pyramidal neurons in the CA1 region of the hippocampus also increased, as well as induction of “early genes” required for LTM formation (Fiorenza et al., 2016). The above data are supported by the studies of Hansen et al. (Hansen et al., 2010) and Siegert et al. (Siegert et al., 2015), which showed that overexpression of miR-132 in forebrain neurons in adult mice (transgenic mice), and miR-137 in the dentate gyrus (using the lentiviral technology) leads to LTM impairment.

Contradictory to the above are the studies carried out on aging animals in which the content of many microRNAs decreases with age; however, cognitive impairment occurs (Inukai et al., 2012; Chmielarz et al., 2017). Dicer dysfunction in the cerebellum leads to progressive loss of microRNA and death of Purkinje cells, and in the forebrain it causes abnormal hyper phosphorylation of the tau protein and neurodegeneration similar to that in Alzheimer’s disease, which accordingly impairs cognitive processes (Hébert et al., 2010; Dimmeler, Nicotera, 2013). In addition, impairment of microRNA biosynthesis in dopaminergic neurons due to suppression or depletion of Dicer (tissue-specific inducible suppression) causes dopaminergic cell dysfunction, while pharmacological stimulation is neuroprotective (Chmielarz et al., 2017). In addition, the pharmacological inhibition of Dicer activity with poly-lysine (Poly-L-lysine hydrobromide) disrupts formation of a conditioned reflex with single-trial induced LTM in the mollusk Lymnaea stagnalis (Korneev et al., 2018) and impairs formation of the conditioned defense reflex in the mollusk Helix (Grinkevich, 2019). Thus, the last two studies show that short-term Dicer dysfunction can lead not to improvement, but to impairment of LTM.

As molecular genetic studies continued, it became clear that miRNAs are capable of not only inhibiting LTM, but also improving its formation. Thus, in the lateral amygdala 7 microRNAs upregulated and 32 downregulated by auditory fear training (Griggs et al., 2013). MicroRNAs miR-9 and miR-34 do not suppress, but support the capacity of spatial learning (the Morris water maze) and reference memory, respectively (Malmevik et al., 2016). The expression of a number of microRNAs in the hippocampus is activated during contextual fear formation (Vetere et al., 2014; Jovasevic et al., 2015). Thus, it became clear that the effect of microRNA on cognitive processes can be multidirectional, which was shown in numerous further studies.

## MicroRNAs that negatively regulate LTM formation

The best-studied microRNAs whose expression decreases during learning include miR-124, miR-134, and miR-206.

miR-124 is one of the first studied microRNAs associated with LTM formation. It is a highly conserved microRNA with a high level of expression in the central nervous system. In 2009, a comprehensive work was published, which for the first time demonstrated involvement of miR-124 in LTM formation and studied its function (Rajasethupathy et al., 2009). As a learning model, the authors used long-term facilitation of synaptic connection between sensory and motor neurons of the mollusk Aplysia. As noted above, facilitation is an essential component in formation of a number of conditioned reflexes, including defensive ones, and is successfully used in the studies of the mechanisms of LTM formation (Kandel, 2012). It was shown that during development of facilitation, the level of miR-124 decreases, and, accordingly, translation of the target of miR-124, the transcription factor CREB-1, is activated (Rajasethupathy et al., 2009). As a result, CREB-1-dependent induction of the genes involved in synaptic modifications takes place, leading to a long-term increase in efficiency of synaptic transmission. At the same time, regulation of miR-124 expression is effected by the modulatory mediator serotonin, which mediates the action of the sensitizing pain stimulus, through PKA-MAPK/ERK-dependent signaling cascades (Rajasethupathy et al., 2009)

Further studies showed that miR-124 also plays an important role in LTM formation in vertebrates, in which, similarly to Aplysia, miR-124 is inhibited during learning (Yang et al., 2012; Malmevik et al., 2016). For example, the amount of miR-124 decreases in the hippocampus during spatial learning and social interactions in mice (Yang et al., 2012). In this case, the target of miR-124 is the transcription factor Zif 268, which takes an active part in cognitive processes; accordingly, a decrease in the miR-124 amount induces translation of Zif 268 (Yang et al., 2012). Increased expression of miR-124 (vector rAAV1/2-miR-124), or knockdown of Zif 268 (LNA-Zif268 antisense) have a negative effect on LTM, and knockdown of miR-124 (LNAmiR-124 antisense) restores expression Zif268 and reverses of LTM formation

The expression of miR-124 is regulated through cAMP and its intracellular receptors EPAC1 and EPAC2. Moreover, in EPAC–/– mice, impairment of spatial learning and memory, as well as social interactions, suppression of synaptic transmission, and impairment of long-term posttetanic potentiation in the hippocampus are observed (Yang et al., 2012). It has been shown that inhibition of miR-124 in hippocampal neurons leads to improvement in LTM, potentially through an increase in the level of expression of genes associated with synaptic plasticity and neuronal transmission (Malmevik et al., 2016). At the same time, genes associated with translation and neurodegenerative diseases are suppressed.

A decrease in the miR-124 expression level associated with improvement in LTM formation in mice was noted in the work of Konopka et al. (Konopka et al., 2010). miR-124 is also involved in memory consolidation during sleep (Karabulut et al., 2019). Post-learning sleep deprivation during specific time windows induces expression of miR-124 in the hippocampus, inhibits synthesis of the neurotrophic factor BDNF, which is the target of miR-124, and, accordingly, disrupts LTM consolidation.

miR-134 is highly expressed in the brain and is detected not only in the bodies of neurons, but also in dendrites (Bicker et al., 2013). As in the case of miR-124, overexpression of miR-134 (the lentiviral technology) in the CA1 region of the hippocampus leads to significant deterioration in LTM formation in the contextual fear-conditioning paradigm and to abrogated long-term potentiation in this structure (Gao et al., 2010). miR-134, like miR-124, affects synaptic plasticity through post-transcriptional regulation of CREB-1 and BDNF in a CREB-dependent way. In turn, miR-134 expression is regulated by SIRT1 deacetylase. In mutant mice lacking the catalytic activity of SIRT1 in the brain, an increase in levels miR-134 is observed, followed by repression of target genes, and, accordingly, impairment of LTM (Gao et al., 2010). The increase in miR-134 levels is sufficient to mimic the behavioral and electrophysiological phenotypes of SIRT1-deficient mice. Conversely, inhibition of miR-134 reverses memory in SIRT1 knockdown mice and restores long-term potentiation in the CA1 region of the hippocampus (Gao et al., 2010).

Memory impairment under stress is also associated with suppression of the SIRT1/miR-134 pathway and the downregulation expression of BDNF and synaptic proteins in the hippocampus (Shen et al., 2019). Thus, miR-134 and miR-124 can have a synergistic effect on the expression of genes involved in plastic rearrangements. In addition, disruption of miR-134 and miR-124-dependent regulation is an important mechanism underlying cognitive dysfunction in Alzheimer’s disease (Wang X. et al., 2018; Baby et al., 2020)

miR-206. Increased levels of miR-206 are observed in the brain of Tg2576 mice (a model of Alzheimer’s disease) and in the temporal cortex of the human brain in Alzheimer’s disease (Lee S. et al., 2012). Decreased miR-206 levels lead to improved memory through induction of the neurotrophic factor BDNF. Improving memory through decrease in the content of miR-206-3p in the hippocampus and cortex is also facilitated by administration of donepezil, a drug with an antidementional effect (Wang C. et al., 2017).

Summarizing the above data, down-regulation expression of miR-124, miR-134, and miR-206 is necessary for successful formation of LTM, since these microRNAs normally block the expression of genes the products of which are necessary for plastic rearrangements.

## MicroRNAs positively regulating learning and LTM formation

miR-9-3p positively influences hippocampus-dependent memory. Inhibition of miR-9-3p in the hippocampus leads to impairment of long-term post-tetanic potentiation (LTP) and disruption of LTM through increased expression of Dmd (dystrophin) and SAP97 (synapse-associated protein 97) genes, which are negatively correlated with LTP (Sim et al., 2016). At the same time, miR-9-5p, which is formed from a common precursor, is not involved in these processes. The miR-9 family is also involved in the regulation of synaptogenesis during early brain development, and a link was found between these developmental events and cognitive functions later in the adult life (Lin et al., 2017)

miR-92. The level of miR-92 increases in the hippocampus during the contextual fear memory formation in mice, which reduces expression of several miR-92 targets, including proteins KCC2, CPEB3 and MEF2D, which negatively regulates memory-induced structural plasticity (Vetere et al., 2014). Selective inhibition of miR-92 in CA1 neurons of the hippocampus (lentiviral technology) leads to upregulation of KCC2, CPEB3 and MEF2D, prevents the learning-induced increase in the spine density and impairs this type of memory.

miR-195. Overexpression of miR-195 in the rat hippocampus using lenti-pre-miR-195 protects against development of dementia, and its inhibition (knockdown by antisense microRNA – lenti-pre-AMO-miR-195) leads to impairment of spatial memory (Morris water maze) (Ai et al., 2013). Potential targets for miR-195 are APP and BACE1 proteins associated with β-amyloid aggregation.

MicroRNA cluster miR-183/96/182. Enhanced expression of the microRNA cluster miR-183/96/182 in the hippocampus promotes LTM formation (an object recognition task), and miR-183/96/182 expression is regulated by protein phosphatase PP1 (Woldemichael et al., 2016). An increase in the levels of miR-183/96/182 leads to suppression of histone deacetylase HDAC9 activity and promotes LTM formation. It is known that HDAC9 negatively affects LTM through deacetylation of histones and chromatin remodeling (Grinkevich, 2012; Fischer, 2014). Downregulation of the miR-183/96/182 cluster leads to memory impairment in the old age, and memory can be improved by overexpression of this cluster (Jawaid et al., 2019).

**Fig. 1.**
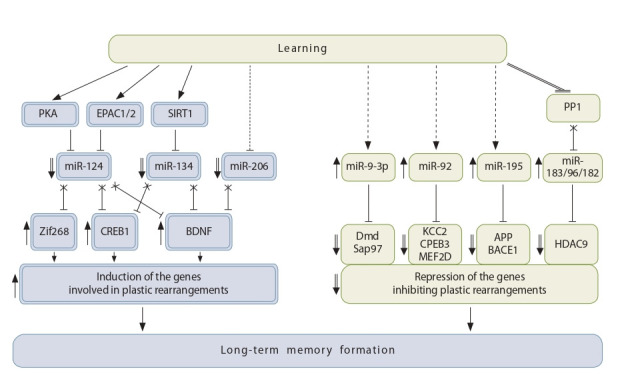
MicroRNA-dependent regulation of gene expression in the long-term memory formation. During training, both inhibition of expression of a number of miRNAs (miR-124, miR-134, miR-206 – marked with a double line) and activation (miR-9-3p, miR-92, miR-195 and cluster miR-183/96/182 – marked with a single line) can occur. Downregulation of miRNAs is needed to reactivate genes, the products of which are necessary for plastic rearrangements and are regulated by transcription factors Zif268, CREB1 and by the growth factor BNDF (all marked with a double line). MicroRNAs which are activated during training repress mRNAs encoding proteins that inhibit structural and synaptic plasticity (Dmd, Sap97, KCC2, CPEB3 and MEF2D), proteins promoting β-amyloid aggregation (APP and BACE1) and also proteins repressing synthesis of histone deacetylase HDAC9 (all marked with a single line). PKA – protein kinase A; EPAC1/EPAC2 – intracellular cAMP receptors; SIRT1-deacetylase and PP1-protein phosphatase are involved in the regulation of microRNAs shown in the diagram. The pathways of regulation of microRNAs that are not described in the literature analyzed in this review are indicated by dashed lines.

Thus, miRNAs (miR-9-3p, miR-92, miR-195 and the miR-183/96/182 miRNA cluster), which contribute to LTM formation, repress mRNAs encoding proteins that inhibit LTP (Dmd, Sap97), structural plasticity (KCC2, CPEB3, MEF2D) and gene silencing (histone deacetylase HDAC9), as well as proteins causing β-amyloid aggregation (APP and BACE1). Below is a simplified diagram of microRNA-dependent regulation of long-term memory formation (Fig. 1).

## MicroRNAs that can influence learning and long-term memory in both positive and negative ways

On the other hand, to date, a large number of miRNAs have been described that can influence formation of LTМ in both positive and negative ways.

miR-132. CREB1-dependent activation of miR-132 expression in the hippocampus is observed during formation of fear-induced memory, matches the kinetics of inducing immediate early genes and regulates the spine size in the presynapses (Nudelman et al., 2010). While in transgenic mice (tTA::miR132), with increased expression of miR-132 in forebrain neurons, violation of LTM formation is observed (Hansen et al., 2010). It should be noted that in this case, another type of learning was studied – recognition of new objects. Nevertheless, the authors note that, in tTA::miR132 transgenic mice, there is a decrease in the expression of MeCP2, a protein involved in development of the Rett syndrome and other mental disorders, as well as a noticeable increase in dendritic spine density in the hippocampus, which ultimately should lead to improving LTM. The authors associate all these inconsistencies with too high expression of miR-132.

miR-34. The data on the effect of the miR-34 family, consisting of three members, miR-34a, miR-34b, and miR-34c, on the learning of microRNAs are even more diverse. It has been shown that inhibition of all the members of miRNAs of this family in hippocampal neurons with using AAVdelivered miRNA sponges reduces the ability for reference memory and causes transcriptome changes associated with transduction of neuroactive ligand-receptors and cell communication (Malmevik et al., 2016). Inhibition of miR-34a alone has a similar effect on LTM in rats. In this case, we are talking about amygdala-dependent memory (auditory fear conditioning) (Dias et al., 2014). The learning-induced increase in miR-34a amount in the basolateral amygdala suppresses the Notch pathway. Given the leading role of the Notch pathway proteins in embryonic development and synapse maturation, the authors believe that Notch signaling normally maintains the steady state of synaptic stability by suppressing synaptic plasticity. Fear-mediated, transient increases in miR-34a in the amygdala reduce Notch signaling, thereby creating an environment that temporarily allows synaptic modification and hence long-term memory consolidation

Unlike miR-34a, miR-34c is a negative factor in memory consolidation. The level of miR-34c increases in the hippocampus during aging, which contributes to impairment of learning and memory, can be restored by inhibiting this microRNA (Zovoilis et al., 2011). In this study, the effect of miR-34c was attributed, at least in part, to a decrease in the target of miR-34c deacetylase SIRT1. The content of miR-34c is also increased in the hippocampus in patients with Alzheimer’s dementia and in hippocampal neurons in APPPS1-21 transgenic mice exhibiting β-amyloid pathology and cognitive deficits at an early age (Zovoilis et al., 2011). In turn, SIRT1 modulates synaptic plasticity and memory formation through a miR-134-mediated mechanism (Gao et al., 2010). Thus, miR-34a is positively associated with amygdala-dependent LTM, while miR-34c is negatively associated with hippocampus-dependent LTM.

miR-137. This microRNA is being intensively studied both in connection with the mechanisms of LTM and with various cognitive pathologies, and its functions are very diverse. It has been shown that activation of miR-137 is required for long-term memory formation in the pond snail Lymnaea (Korneev et al., 2018). At the same time, miR-137 inhibits the transcription factor CREB2, a negative regulator of the expression of genes necessary for LTM formation. In mice knocked out of the miR-137 gene, spatial learning and memory are impaired, which is potentially associated with increased expression of the Ezh2 gene (Yan et al., 2019). Ezh2 encodes histone-lysine N-methyltransferase involved in the methylation of histones at sites that inhibit gene expression. Thus, it has been shown that miRNAs are capable of modulating another regulatory pathway, namely, epigenetic chromatin remodeling. Data obtained in heterozygous mice with partial loss of miR-137 function support the activating effect of miR-137 on LTM (Cheng et al., 2018). On the other hand, overexpression of miR-137 in the dentate gyrus of the hippocampus disrupts presynaptic plasticity and impairs fear-induced context memory, while the expression of presynaptic target genes associated with the release of synaptic vesicles (complexin-1, Nsf, and synaptotagmin-1) decreases (Siegert et al., 2015). miR-137 is also implicated in the Pb-induced hippocampus-dependent spatial memory impairment (Gu X. et al., 2019). It was shown that chronic oral administration of lead acetate (PbAc) with drinking water causes a change in the genomic landscape of histone H3 methylation at the H3K27me3 site in the hippocampus. It should be noted that the change in methylation is associated with activated interaction of miR-137 and EZH2 methyltransferase, which make up a mutually inhibitory loop. Overexpression of EZH2 in PbAc-treated rats reverses H3K27me3 methylation and partially restores spatial memory

miR-153 also has a multidirectional effect on LTM. Thus, the expression of miR-153 is specifically induced in the hippocampus during fear-dependent memory acquisition (Mathew et al., 2016). At the same time, miR-153 inhibits the expression of key components of the vesicular transport system, reduces the level of the glutamate receptor A1 trafficking and neurotransmitter release. On the other hand, knockdown of miR-153 in the hippocampus of adult mice leads to improvement in the fear memory. The authors explain the resulting contradiction by the fact that miR-153, along with, possibly, other fear-induced miRNAs, acts as a component of a feedback loop that blocks neuronal hyperactivity by inhibiting the vesicular transport pathway (Mathew et al., 2016). Dysregulation of miR-153 has been associated with decreased learning and memory ability in autistic mice (You et al., 2019). It has been shown that the target of miR-153 is the LEPR (a leptin receptor) and the JAK-STAT signaling pathway regulated by it. Overexpression of miR-153 suppresses LEPR and the JAK-STAT signaling pathway, which leads to an increase in BDNF expression, an increase in the proliferative capacity of hippocampal neurons, and promotes LTM formation. That is, the high expression of miR-153, and not its knockdown, as stated in the work of Mathew et al. (Mathew et al., 2016), improves LTM formation.

miR-182. An increase in the expression of this miRNA in the hippocampus within the miR-183/96/182 miRNA cluster promotes the hippocampus-dependent LTM formation (Woldemichael et al., 2016). On the contrary, in the amygdala, during formation of amygdala-dependent LTM, a decrease in the amount of miR-182 is noted, and its artificial overexpression leads to a disruption of LTM (Griggs et al., 2013). The miR-182 targets in the amygdala are the key actin-regulating proteins, cortactin and Rac1. Interestingly, another member of the miR-183/96/182 cluster, miR-96, is not expressed in the amygdala. The mechanisms of independent functioning of some microRNAs belonging to clusters are currently not clear, but presumably, this phenomenon is associated with the type of cells in which their differential expression occurs (Banks et al., 2020).

Thus, miR-132, miR-34, miR-137, miR-153, and miR-182 may influence LTM formation in both positive and negative ways, depending on the learning paradigm and the brain structures involved in learning. In addition, the effect of microRNAs on memory may often depend on their concentration. Thus, a moderate increase in miR-212/132 facilitates, and an excessive increase in its expression negatively affects, learning and memory (Benito et al., 2018). The important role of miRNAs in LTM formation is also evidenced by the recent studies related to sleep deprivation.

## MicroRNAs, long-term memory and sleep

It is widely known that even a short period of sleep deprivation may impair memory formation. Sleep disturbance, caused by emotional overload, the rugged rhythm of life and chronic life stress, causes a decrease in performance and cognitive functions in a significant number of the world population. It is believed that one of the mechanisms of the effect of sleep deprivation on cognitive processes may be impaired epigenetic regulation of gene expression, including genes associated with microRNA dysfunction (Gaine et al., 2018).

Thus, the recent studies have shown that sleep deprivation significantly changes the profiles of DNA methylation and, accordingly, the synthesis of RNA, including microRNA (Nilsson et al., 2016). On the other hand, miRNAs are involved in the regulation of circadian rhythms that regulate the sleep and wakefulness cycles (Gaine et al., 2018). Disruption of microRNA biogenesis may lead to changes in the circadian rhythms and potentially affect cognitive abilities. In patients with depression and late insomnia, genetic variants of miR-182 miRNA were found that induce inhibition of expression of circadian clock proteins CLOCK and DSIP (Saus et al., 2010). In addition, impaired expression of miR-182, along with miR-132 and miR-124, is observed during the paradoxical sleep phase deprivation and leads to disruption of hippocampus-dependent LTM (Karabulut et al., 2019). At the same time, the synthesis of the growth factor BDNF, which is involved in memory consolidation during sleep, changes markedly (Karabulut et al., 2019). The relation of these microRNAs with numerous cognitive processes has been described in the chapters above. In addition, miR-132 is a key pathway for coupling the circadian rhythm and the rhythm of cognitive abilities (Aten et al., 2018).

Sleep deprivation also disrupts the content of miRNAs let-7b, miR-125a, and miR-138 (Gaine et al., 2018). It is believed that induction of the epigenetic processes caused by sleep deprivation is carried out by signaling cascades that regulate synaptic plasticity (Havekes, Abel, 2017). Sleep disturbance is common in people with fear-related anxiety disorders. It has been shown that some microRNAs, such as miR-132 and miR-144-3p, play an important role both in generation of fear and in suppression of memories of it and are associated with consolidation and reconsolidation of LTM, respectively (Murphy, Singewald, 2018). At the same time, impaired miR-132 expression is observed during sleep deprivation and is accompanied by cognitive dysfunctions (Karabulut et al., 2019).

## Perspectives for improving cognitive processes by influencing microRNA biogenesis

In recent years, more and more data have been accumulated that microRNAs play an important role in cognitive disorders in neurological, neurodegenerative, and age-related dysfunctions (Ramakrishna, Muddashetty, 2019; Wingo et al., 2020; Wu, Kuo, 2020). Coverage of these issues requires a separate review. However, it is important to note that there is an increasing number of predictions regarding the possibility of therapeutic treatment of a number of cognitive impairments by influencing microRNA biogenesis (Cao, Zhen, 2018; Paul et al., 2020; Wingo et al., 2020). Optimism in this area is determined by the emergence of new genome editing technologies using the CRISPR/Cas9 system adapted to microRNA (Aquino-Jarquin, 2017). In addition, CRISPRCas9 systems have been developed, which allows editing of the genome in a specific cell population without affecting other organs and tissues (Hirosawa et al., 2017; Hoffmann et al., 2019). These technologies are especially important in studying the mechanisms of the central nervous system functioning and the prospects for therapeutic intervention in the pathogenesis of brain diseases.

**Fig. 2.**
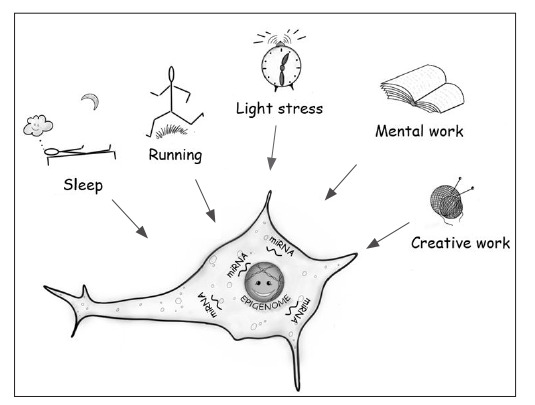
Ways to improve cognitive abilities.

On the other hand, it has been known for a long time that cognitive processes can be improved through intensification of cognitive processes, fine motor movement work or physical exercise. In recent years, it has been shown that mental and physical activities improve the epigenetic processes involved in formation of LTM and protect neurons from death. For example, running exercise helps to improve memory in mice with traumatic brain injury (Hu T. et al., 2015). At the same time, there is a decrease in the content of miR-21 and an increase in the number of branch points of the hippocampal neurons. Physical exercise also improves the cognitive function in aged mice (Jessop, Toledo-Rodriguez, 2018). The process involves miR-137, which is associated with good memory and neurogenesis in adults (the rate of neurogenesis decreases with age). In addition, the possibility of improving aging-related memory decline by enriching the environment (a combination of cognitive training and physical exercise) has been shown (Jawaid et al., 2019). In this case, an increase in the biogenesis of the miR-183/96/182 cluster is stimulated, which is closely associated with hippocampus-dependent memory. Environmental enrichment also attenuates mild cognitive impairment by activating the SIRT1/miR-134 signaling pathway in the hippocampus, followed by ultrastructure changes of synapses and dendritic remodeling (Shen et al., 2019). Moreover, it has been shown that an environmental enrichment is capable of enhancing synaptic plasticity and cognition even in the next generation, with sperm RNA, and especially miRs 212/132, mediating the effect (Benito et al., 2018). Widely available ways to improve cognitive abilities are shown in Fig. 2.

Mental work, physical exercise, manual creativity, light stress, good sleep and a good mood are able to protect neurons from death and improve cognitive processes via epigenetic mechanisms on chromatin remodeling and miRNA expression.

## Conclusion

Thus, miRNAs are widely involved in the regulation of gene expression required for the long-term memory formation. Further study of ways to regulate microRNA activity in individual cell populations, as well as detailed study of their targets using bioinformatics analysis methods, will help to better understand the molecular genetic basis of long-term memory and potentially to develop methods of treatment in case of cognitive dysfunctions in neurodegenerative pathologies and senile dementia

## Conflict of interest

The authors declare no conflict of interest.
